# Proximitomics by Reactive Species

**DOI:** 10.1021/acscentsci.4c00373

**Published:** 2024-06-12

**Authors:** Shaoran Zhang, Qi Tang, Xu Zhang, Xing Chen

**Affiliations:** † College of Chemistry and Molecular Engineering, Peking University, Beijing 100871, People’s Republic of China; ‡ Peking-Tsinghua Center for Life Sciences, Peking University, Beijing 100871, People’s Republic of China; § Beijing National Laboratory for Molecular Sciences, Peking University, Beijing 100871, People’s Republic of China; ∥ Synthetic and Functional Biomolecules Center, Peking University, Beijing 100871, People’s Republic of China; ⊥ Key Laboratory of Bioorganic Chemistry and Molecular Engineering of Ministry of Education, Peking University, Beijing 100871, People’s Republic of China

## Abstract

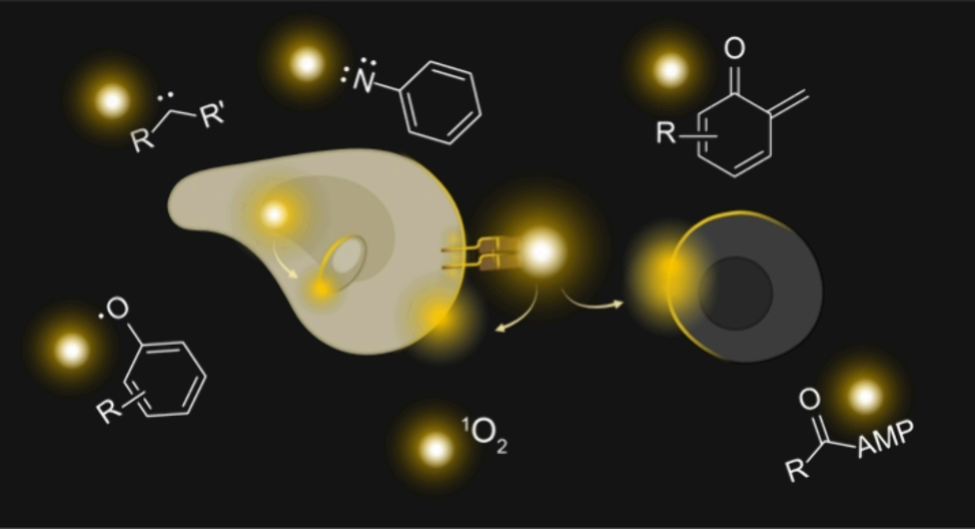

The proximitome is defined as the entire collection of biomolecules spatially in the proximity of a biomolecule of interest. More broadly, the concept of the proximitome can be extended to the totality of cells proximal to a specific cell type. Since the spatial organization of biomolecules and cells is essential for almost all biological processes, proximitomics has recently emerged as an active area of scientific research. One of the growing strategies for proximitomics leverages reactive species—which are generated in situ and spatially confined, to chemically tag and capture proximal biomolecules and cells for systematic analysis. In this Outlook, we summarize different types of reactive species that have been exploited for proximitomics and discuss their pros and cons for specific applications. In addition, we discuss the current challenges and future directions of this exciting field.

## Abstract 2

Proximitomics leverages reactive species to chemically tag and capture proximal biomolecules and cells for systematic analysis, uncovering their interactions and spatial organizations in complex biological systems.

## 1 Introduction

“*A living organism feeds upon negative entropy*”. This famous quote comes from “What is Life” by Erwin Schrodinger in 1944, where he stated that life is continually maintained at a fairly low level of entropy. From a biochemical perspective, this concept is well-evidenced by the fact that life consumes energy to exist in a spatially organized form at levels ranging from biomolecules to cells, tissues, and the entire organism. At the molecular level, the spatial organization of biomolecules within a cell dictates their interactions to form a sophisticated network, which governs diverse biochemical processes. For example, the subcellular localization of proteins and RNAs is tightly regulated to ensure proper functions.^1,2^ Zooming out to the cellular level, cells communicate with neighboring cells to coordinate cellular behaviors and form functional tissues and organs.^3^ The spatial organization of cells determines how these intercellular communications occur, underscoring its essential roles in maintaining cellular functions within a multicellular organism.^4^ Given the importance of spatial organization of biomolecules and cells, methods for identifying the proximitome, which is defined as the entire collection of biomolecules in the proximity of a target biomolecule or cells in the proximity of a specific cell type, are currently of great research interest.

**Figure 1:**
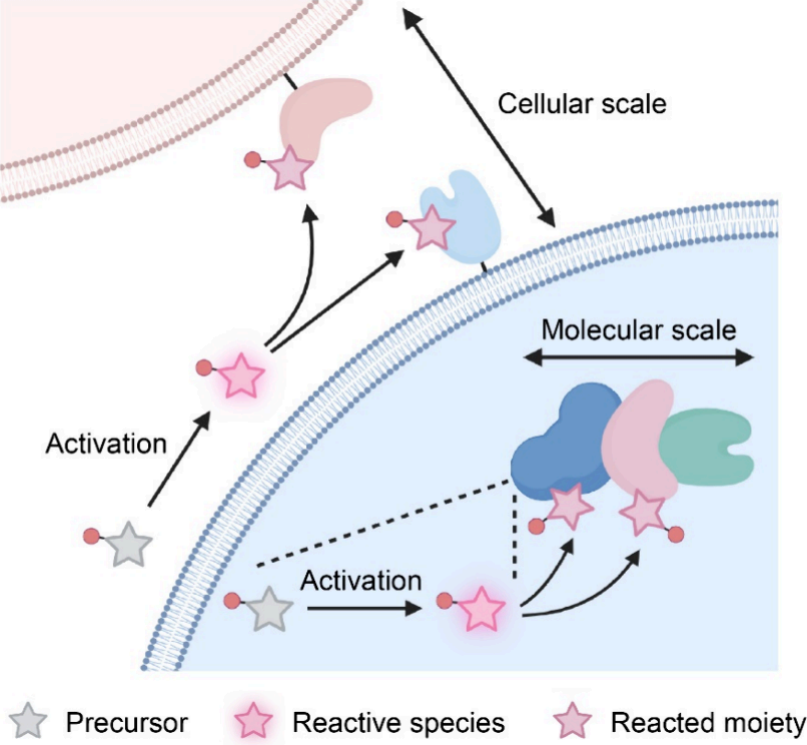
Schematic depicting proximitomics based on proximity labeling. Precursors are activated to reactive species, which covalently tag nearby biomolecules or cells for subsequent analysis on both molecular and cellular scales.

Toward proximitomics, one promising approach is to exploit reactive species, which can be generated locally and confined spatially to chemically tag proximal biomolecules and cells (Figure 1). The generation of reactive species occurs only at the positions of biomolecules or cells of interest and rapid quenching of the reactive species by water and surrounding molecules confines the labeling within the diffusion distance (typically from nanometers to micrometers). Subsequently, isolation of the tagged biomolecules or cells enables downstream analysis, such as proteomic identification and single-cell RNA sequencing (scRNA-seq). Accordingly, a variety of methods for proximity labeling have been developed for the past decade, with an array of chemical species having been successfully introduced into proximity labeling, including phenoxyl radicals, activated carboxylates, singlet oxygen, carbenes, nitrenes, and quinone methides.^5−7^ These reactive species and the corresponding labeling methods vary in multiple aspects, calling for careful comparison and evaluation before use. For example, the reactive species can be activated by various means, such as using a genetically encoded enzyme or an exogenously added photocatalyst, each tailored to specific applications. Furthermore, the half-life and thus the labeling radius differ between these reactive species, making them suitable for proximitomics on different scales. In addition, these reactive species exhibit distinct reactivity toward different types of biomolecules. As a result, the intricate interplay of these factors underscores the importance of choosing the proper proximity labeling methods in different scenarios.

In this Outlook, we discuss recent advancements in proximitomics with an emphasis on the properties of reactive species. Some of the biological applications of proximitomics are also highlighted. Instead of comprehensively covering all of the important works, we mainly focus on the chemical insights of proximity labeling, hoping to provide some inspiration for further advancing this exciting field.

## 2 Reactive Species Used in Proximitomics

### 2.1 Phenoxyl Radicals

**Figure 2:**
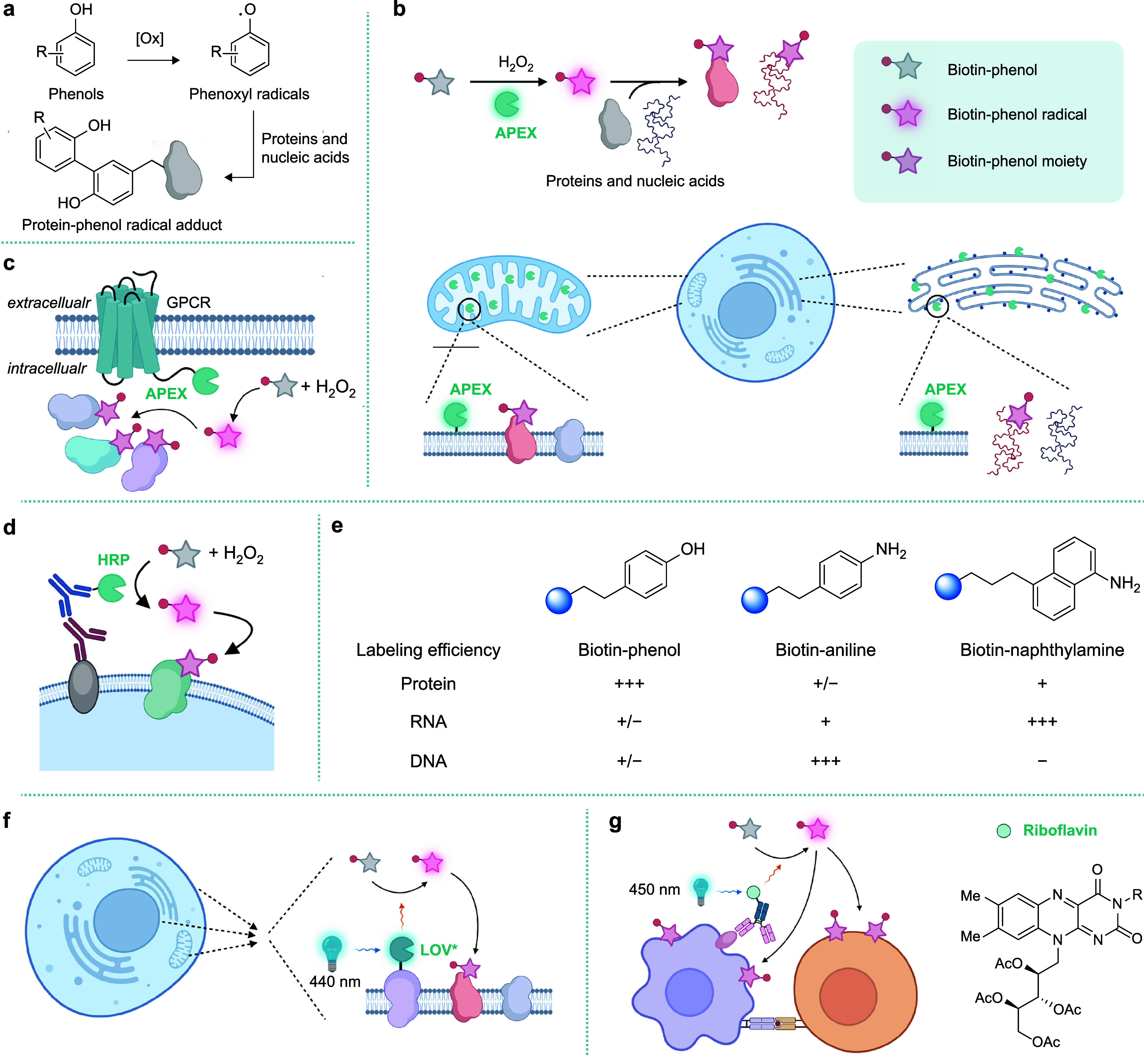
Proximitomics based on phenoxyl radicals. (a) Upon generation by phenol oxidation, phenoxyl radicals react with proteins and nucleic acids. For proteins, tyrosine residues are the main substrates for phenoxy radicals. (b) APEX catalyzes the oxidation of biotin–phenol into phenoxyl radicals by H_2_O_2_, thus allowing for spatial proteomics and transcriptomics analysis in various subcellular compartments. (c) APEX enables the identification of protein interaction networks, e.g., intracellular regulators of the GPCR signaling pathway. (d) Directing HRP by antibody–antigen recognition into specific subcellular regions in fixed cells or on the surface of living cells enables proximitomic studies without genetic manipulations. (e) Compared to biotin–phenol, biotin–aniline and biotin–naphthylamine are identified as better probes for APEX-based nucleic acid labeling. (f,g) Flavin-based photocatalysts enable the photochemical activation of phenol into phenoxyl radicals using oxygen as the oxidant. Recombinant LOV variants and antibody–flavin conjugates are used for protein proximity labeling within living cells (f) and recording cell–cell interactions events (g), respectively.

Phenols can be oxidized into phenoxyl radicals by various oxidants (Figure 2a). It has long been known that phenoxyl radicals can react with proteins and DNAs, forming phenol–protein and phenol–DNA adducts.^8,9^ For example, tyrosyl radicals are documented to induce protein–protein and protein–DNA cross-linking.^10^ Tyrosine residues are the main substrates on proteins that react with phenoxyl radicals, leading to a tyrosine–phenol linkage between two ortho carbons (Figure 2a).^11^ As the half-life of phenoxyl radicals is typically less than 1 ms,^12^ precise control of phenoxyl radical formation would enable proximity labeling. This concept was first demonstrated in 1990s by the Litt group with the utilization of horseradish peroxidase (HRP)-conjugated antibodies to catalyze the oxidation of biotin–phenol by H_2_O_2_.^13^ The resulting phenoxyl radicals covalently label molecules proximal to the antigen so that the immunosignals are amplified via this process. However, many peroxidases, including HRP, contain essential disulfide bonds and therefore fail to function in the cytosol, making applications of this strategy in living cells challenging.

In 2012, the Ting group reported an engineered ascorbate peroxidase (APEX) that functioned intracellularly as a genetically encoded reporter for electron microscopy (EM).^14^ Recombinant expression of APEX in mammalian cells enables the initiation of polymerization of 3,3′-diaminobenzidine (DAB) using H_2_O_2_ as the oxidant, yielding EM contrast upon treatment with aqueous OsO_4_. Similar to HRP, APEX is capable of oxidizing a variety of aromatic compounds including phenols. With APEX and biotin–phenol, the same group demonstrated protein proximity labeling in living cells (Figure 2b).^11^ By expressing APEX in specific subcellular compartments followed by treating cells with H_2_O_2_ and biotin–phenol, subcellular proteins were biotinylated within only 1 min and purified for proteomic analysis. Applying this method, proteins localized in the mitochondrial matrix were identified with over 95% specificity. By introducing one more mutation in the APEX enzyme, the Ting group further developed APEX2 with improved enzyme activity and labeling efficiency.^15^

The APEX/APEX2-based proximity labeling methods have soon been used for a number of biological studies.^16^ For example, the Kruse group employed APEX coupled with isobaric tagging and mass spectrometry to quantitatively monitor GPCR agonist response in living cells (Figure 2c).^17^ Of note, although HRP is dysfunctional in the cytosol, its activity in oxidative environments, such as the secretory pathway and extracellular regions, is higher than APEX. As a result, HRP serves as a better option for proximity labeling in these compartments. For instance, the expression of a plasma membrane-tethered HRP in neurons allows for the identification of the synaptic proteome.^18^ Additionally, HRP conjugated with antibodies or lectins enables the profiling of protein–protein interaction in intracellular regions of fixed cells or on the surfaces of living cells without the need of genetic manipulations (Figure 2d).^19,20^

In addition to proteins, nucleic acids also serve as good substrates for phenoxyl radicals. The Ting group discovered that the phenoxyl radicals primarily react with guanosines in RNA, leading to the development of APEX-seq for subcellular transcriptome profiling (Figure 2b).^21^ This chemistry also enabled two approaches using antibody–HRP in fixed cells and recombinant expression of APEX2 in living cells, respectively, for DNA labeling and mapping of 3D genome organization.^22,23^ Given that phenoxyl radicals preferentially react with electron-rich amino acid residues and thereby may not be the optimal structure for nucleic acid labeling, the Zou group screened a panel of aromatic compounds and identified the biotin–aniline as a novel probe with significantly higher reactivity toward nucleic acids (Figure 2e).^24^

One limitation of APEX/HRP-based proximitomics is the cytotoxicity caused by H_2_O_2_. To overcome this issue, photocatalysts have recently been developed for catalyzing the generation of phenoxyl radicals using oxygen as the oxidant. For example, flavin cofactors were identified as effective photosensitizers for phenol oxidation.^25^ The LOV (Light-Oxygen-Voltage) domains of *Arabidopsis* phototropin are a family of flavin mononucleotide (FMN)-binding proteins.^26^ Recently, proximity labeling in living cells has been demonstrated by recombinant expression of an engineered LOV domain for generating phenoxyl radicals from phenols upon blue light irradiation (Figure 2f).^27^ In addition, the antibody–flavin conjugates that bind specific cells have been utilized as photocatalysts for recording cell–cell interactions between B cells and T cells (Figure 2g).^28^ This method, termed PhoTag, was the first to employ phenoxyl radicals for proximity labeling on the cellular scale. Nevertheless, rapid quenching of phenoxyl radicals results in the labeling primarily occurring at the cell–cell interface, so that only contact-dependent cell–cell interaction events were recorded.

### 2.2 Activated Carboxylates

**Figure 3:**
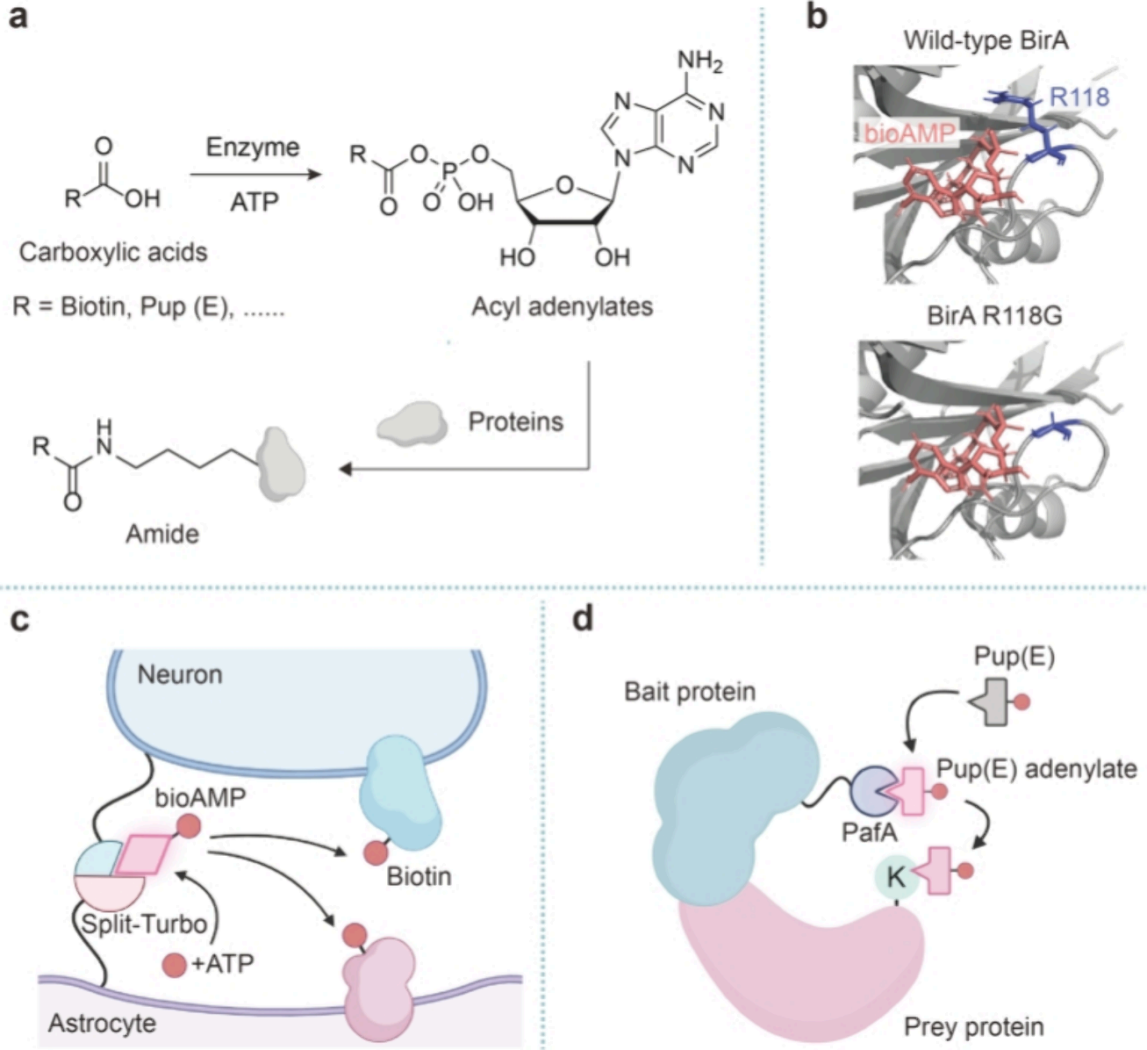
Proximitomics based on activated carboxylates. (a) Carboxylates are enzymatically activated into acyl adenylates, which react with amines on proteins to form amides. (b) Crystal structures (PDB code: 4WF2) of wild-type BirA and its promiscuous variant (BirA^R118G^). The bioAMP and key residue R118 are shown in red and blue, respectively. (c) A spilt version of TurboID enables precise labeling of the proteome in the neuron–astrocyte interface. (d) PUP-IT utilizes PafA to catalyze the generation of Pup(E)-adenylates, which in turn covalently tag proximal proteins for subsequent analysis.

Activated carboxylates, including acid anhydrides and activated esters, are widely used reagents for bioconjugation. Unlike the exogenously produced phenoxyl radicals, activated carboxylates are common forms of high-energy molecules involved in a variety of biochemical reactions. For example, amino acids are activated into aminoacyl adenylates by aminoacyl tRNA synthetase followed by conjugation with tRNA.^29^ Similarly, biotin ligase (e.g., BirA in *E. coli*) activates biotin with ATP and generates biotin adenylates (i.e., biotinyl-5′-AMP or bioAMP). The resulting enzyme–bioAMP complex recognizes and biotinylates the lysine residue in the AviTag sequence on protein substrates.^30^ As a mixed acid anhydride, bioAMP displays high reactivity toward primary and secondary amines, making it promising for protein proximity labeling (Figure 3a). To use bioAMP for proximity labeling, a promiscuous variant of biotin ligase that releases bioAMP from the enzyme pocket is desired. Such a mutant (*E. coli* BirA^R118G^) was first identified in 2004 and employed by the Roux group in 2012 to enable the development of BioID (proximity-dependent biotin identification) for protein proximity labeling in living cells (Figure 3b).^31,32^ BioID involves the expression of BirA^R118G^ fused to a bait protein for initiating bioAMP formation in living cells. Prey proteins proximal to the bait protein are thus biotinylated and isolated for subsequent analysis. The user-friendly nature of BioID has contributed to its widespread applications. For example, the Gingras group generated a thorough database for protein subcellular localization in HEK293T cells by using BioID to identify the proximal proteins of 192 protein targets.^33^

Due to the relatively low kinetics of BioID with a typical labeling time of 18–24 h, a series of methods (e.g., BioID2,^34^ BASU,^35^ TurboID,^36^ AirID,^37^ and MicroID2^38^) have subsequently been developed with improved enzyme activity and labeling kinetics (Table 1). TurboID, as one of the exceptional BioID variants developed by the Ting group, possesses 15 mutations compared to the wild-type BirA and requires a labeling time down to 10 min, making it well suited for identifying protein–protein interactions and subcellular proteome with high temporal resolution.^36^ In addition, TurboID also enables applications that require a higher labeling efficiency. For example, TurboID has been recently employed for mapping cell-type-specific secretome in vivo by directing TurboID into the endoplasmic reticulum to label proteins in the secretory pathway.^39,40^

**Table 1:** BioID Variants for Proximity Labeling with Improved Efficiency and Kinetics

enzyme/method	origin	molecular weight (kDa)	engineering	labeling time
BioID^32^	*E. coli*	35	R118G	18–24 h
BASU^35^	*B. subtilis*	29	2 mutations	18 h
BioID2^34^	*A. aeolicus*	27	R40G	16 h
TurboID^36^	*E. coli*	35	15 mutations	10 min
miniTurbo^36^	*E. coli*	28	13 mutations	10 min
AirID^37^	ancestral BirA	37	designed sequence	3 h
MicroID2^38^	*A. aeolicus*	19	7 mutations, C-terminal truncation	3 h

Unlike the proximity labeling methods using phenoxyl radicals, promiscuous biotin ligase activates biotin by using ATP instead of H_2_O_2_, which avoids the introduction of cytotoxicity and thus makes them applicable in vivo. For example, the Soderling group established in vivo BioID (iBioID) in mice and identified the constituents of synaptic protein complexes in the postsynaptic density (PSD) region.^41^ In vivo applications with TurboID were also successfully achieved in *Drosophila*,^42^ mice,^43^ and *Arabidopsis*.^44^ Similar to the APEX/HRP-based strategies, the BioID-based methods are compatible with the extracellular environment. For example, fusing biotin ligase to the ectodomain of transmembrane proteins enabled the identification of protein networks on cell surfaces.^45^ To increase the spatial specificity, a split version of TurboID has been further developed for mapping the perisynaptic cleft proteome specifically in the neuron–astrocyte interface in living mice (Figure 3c).^46^ These works highlight the versatility of bioAMP as a reactive species for proximity protein labeling. Of note, due to the low levels of extracellular ATP, the use of bioAMP in extracellular spaces may require the addition of exogenous ATP.

In addition to bioAMP, the ubiquitin-like system has also been exploited for proximitomics. The ubiquitin system typically involves an E1 enzyme that catalyzes the ATP-dependent adenylation of the C-terminal carboxyl group of the ubiquitin peptide, followed by the generation of E1-ubiquitin in a thioester form.^47^ Subsequent transfer of the ubiquitin peptide to the E2 and E2-E3 complex enables substrate recognition and, finally, protein ubiquitination. Similar to bioAMP, the ubiquitin adenylate and its variants, in principle, can serve as the reactive species for proximity labeling. Along this line, the Zhuang and Wang groups developed the PUP-IT (pupylation-based interaction tagging) method based on PafA, a bacterial E1-like enzyme that adenylates a small peptide substrate Pup(E) (Figure 3d).^48^ Unlike promiscuous biotin ligases, the activated Pup(E) does not diffuse from the pocket of PafA but directly reacts with nearby protein lysine residues in a PafA-bound form, which ensures strict spatial specificity, as only direct binders are labeled by the PUP-IT system. Importantly, both PafA and Pup(E) can be recombinantly expressed, making the whole system genetically encodable.

### 2.3 Singlet Oxygen

**Figure 4:**
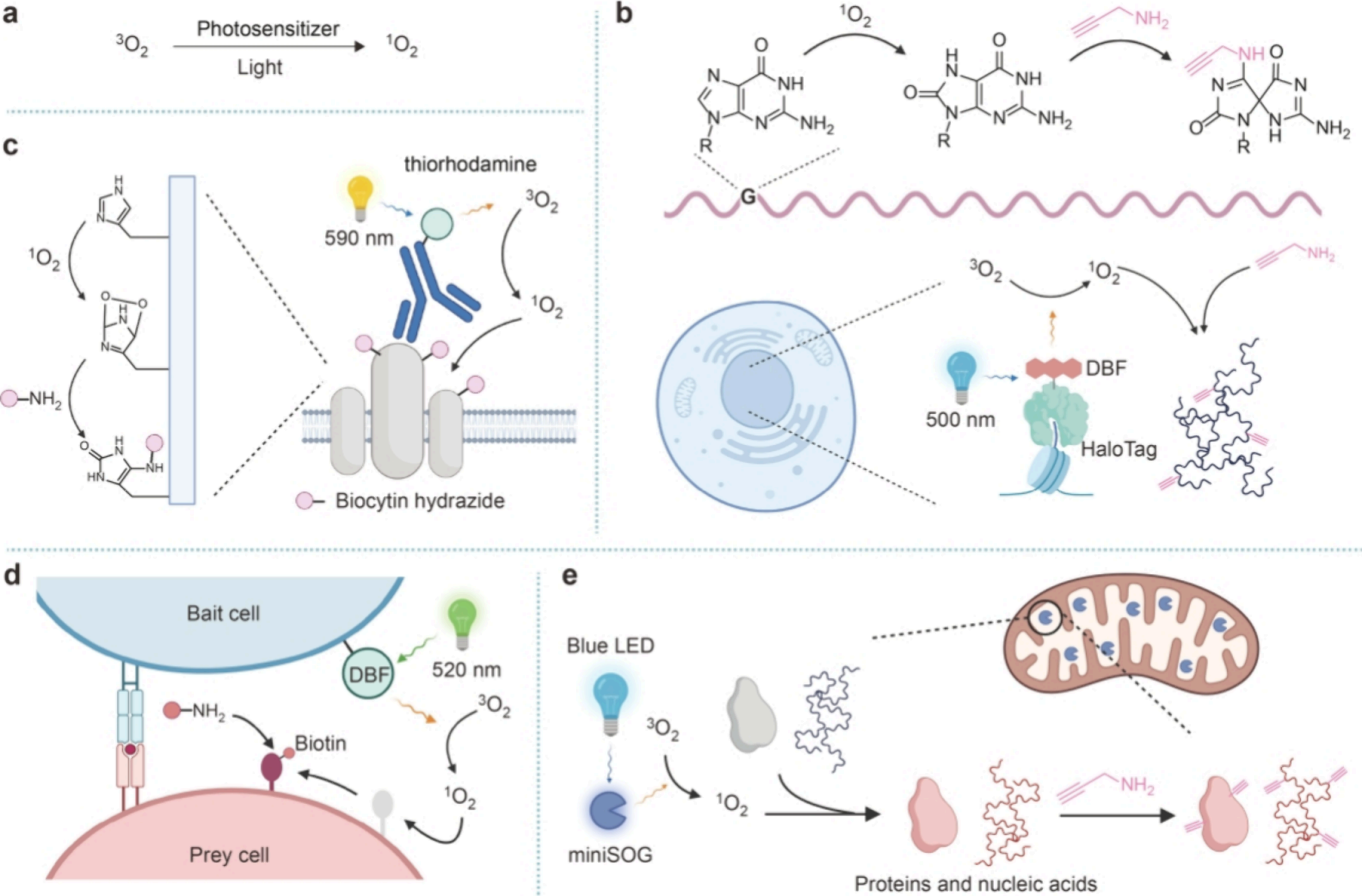
Proximitomics based on singlet oxygen. (a) ^3^O_2_ is converted into ^1^O_2_ upon activation by photosensitizer. (b) DBF is directed to specific subcellular compartments via HaloTag and generates ^1^O_2_ for tagging proximal RNAs upon 500 nm light irradiation. The tagged RNAs are conjugated to alkyne-amine followed by click labeling and subsequent analysis. (c) In LUX-MS, antibody–thiorhodamine conjugates serve as photocatalysts for generating ^1^O_2_ upon 590 nm light irradiation. Proteins proximal to the antigen target are tagged and conjugated with biocytin hydrazide for further enrichment and proteomic analysis. (d) PhoXCELL uses DBF to catalyze ^1^O_2_ formation on cell surfaces. The generated ^1^O_2_ tags both bait cells and interacting prey cells, followed by labeling of tagged cells with alkyne-amine for downstream analysis. (e) Recombinant expression of miniSOG in specific subcellular compartments enables ^1^O_2_-based proximity labeling for proteins, RNAs, and DNAs.

Oxygen molecule normally exists in a triplet form with the unpaired electrons in the same spin state. Upon activation by photosensitizers, triplet oxygen is converted into singlet oxygen (represented as ^1^Δ_g_O_2_, abbreviated as ^1^O_2_) and represents an excited state in which the spin state of one of the unpaired electrons is changed to the opposite orientation (Figure 4a).^49^^1^O_2_ is a strong oxidant and readily oxidizes a variety of biomolecules including lipids, proteins, and nucleic acids.^50^ Since the resulting oxidation products are harmful to the cells, precise control of ^1^O_2_ generation has been used as a strategy for targeted cell ablation and photodynamic therapy.^51,52^ Moreover, ^1^O_2_ generated by small-molecule photosensitizers has also been employed to oxidize DAB for EM.^53^ In these cases, ^1^O_2_ exhibited minimal diffusion in cells, making it a promising reactive species for proximity labeling.

Unlike phenoxyl radicals and activated carboxylates, ^1^O_2_ cannot directly tag the substrates with reporters but instead introduces electrophilic modifications on the biomolecules. Subsequent bioorthogonal conjugation of the oxidation products with an affinity tag allows for enrichment and further analysis. For example, ^1^O_2_ primarily oxidizes guanosine in nucleic acids and produces 8-oxo-guanosine, which can be specifically conjugated to primary amines.^54^ Building on this chemistry, the Spitale group demonstrated the ^1^O_2_-based proximity labeling of RNAs with a small-molecule photosensitizer, dibromofluorescein (DBF) (Figure 4b).^55^ DBF was modified with a HaloTag ligand for targeting specific subcellular compartments. By treating cells with propargyl amine followed by blue light irradiation, subcellular RNAs are tagged with alkynes and further click-labeled with azide–biotin for analysis. In combination with RNA sequencing, the same group developed Halo-seq for quantitative subcellular transcriptome analysis.^56^ Recently, Halo-seq was further used for analyzing RNA distribution across the apicobasal axis in asymmetric cells.^57^ In addition to RNA proximity labeling, ^1^O_2_ also oxidizes histidine into 2-oxo-histidine, which can be conjugated with primary amine probes similar to 8-oxo-guanosine.^58^ By using thiorhodamine as the photosensitizer that was conjugated to antibodies or small-molecule ligands, the Wollscheid group developed LUX-MS for analyzing the cell surfaceome organization at the nanometer scale (Figure 4c).^59^ Both receptors of small molecules and immune synaptic proteomes were precisely identified by LUX-MS. Using a similar chemistry, the Chen and Li groups developed the PhoXCELL (photocatalytic proximity cell labeling) by installing DBF on the cell surface to generate ^1^O_2_ for identifying cell–cell interactions (Figure 4d).^60^ At the cell–cell interface, the generated ^1^O_2_ labeled not only the bait cells but also their interacting cells in proximity. PhoXCELL was applied to identify tumor-antigen-specific T cells within the tumor-infiltrating leukocytes.

The utilization of a small-molecule photosensitizer, however, may cause high background due to nonspecific binding. A fully genetically encoded photosensitizer for ^1^O_2_-based proximitomics is therefore of great interest. Given that the flavin cofactors may catalyze ^1^O_2_ generation, the Tsien group developed such a genetically encoded ^1^O_2_ photocatalyst engineered from the *Arabidopsis* LOV domain, which they termed miniSOG.^61^ The quantum yield of miniSOG reaches 0.47 upon 480 nm light illumination, which is comparable to that of small-molecule photosensitizers. Based on miniSOG, the Zou group developed CAP-seq (chromophore-assisted proximity labeling and sequencing) for subcellular RNA mapping (Figure 4e).^62^ Recombinant expression of miniSOG in different subcellular structures enabled quantitative analysis of RNA localization in various cellular compartments. This strategy was further adopted for DNA and protein labeling with modifications on labeling conditions and the structure of amine probes (Figure 4e).^63−65^ Of note, the generation of ^1^O_2_ by either small-molecule or genetically encoded photosensitizers mainly relies on blue light irradiation, which also activates endogenous photosensitizers such as FMN-binding proteins and thus may introduce background labeling. It was recently discovered that 10 min of exposure to 450 nm light, a typical condition used in the DBF- or miniSOG-based labeling, was sufficient to strongly oxidize over 100 proteins in HeLa and B16F10 cells.^66^ Thus, developing strategies for ^1^O_2_ generation using light with a longer wavelength is a promising future direction.

### 2.4 Carbenes and Nitrenes

**Figure 5:**
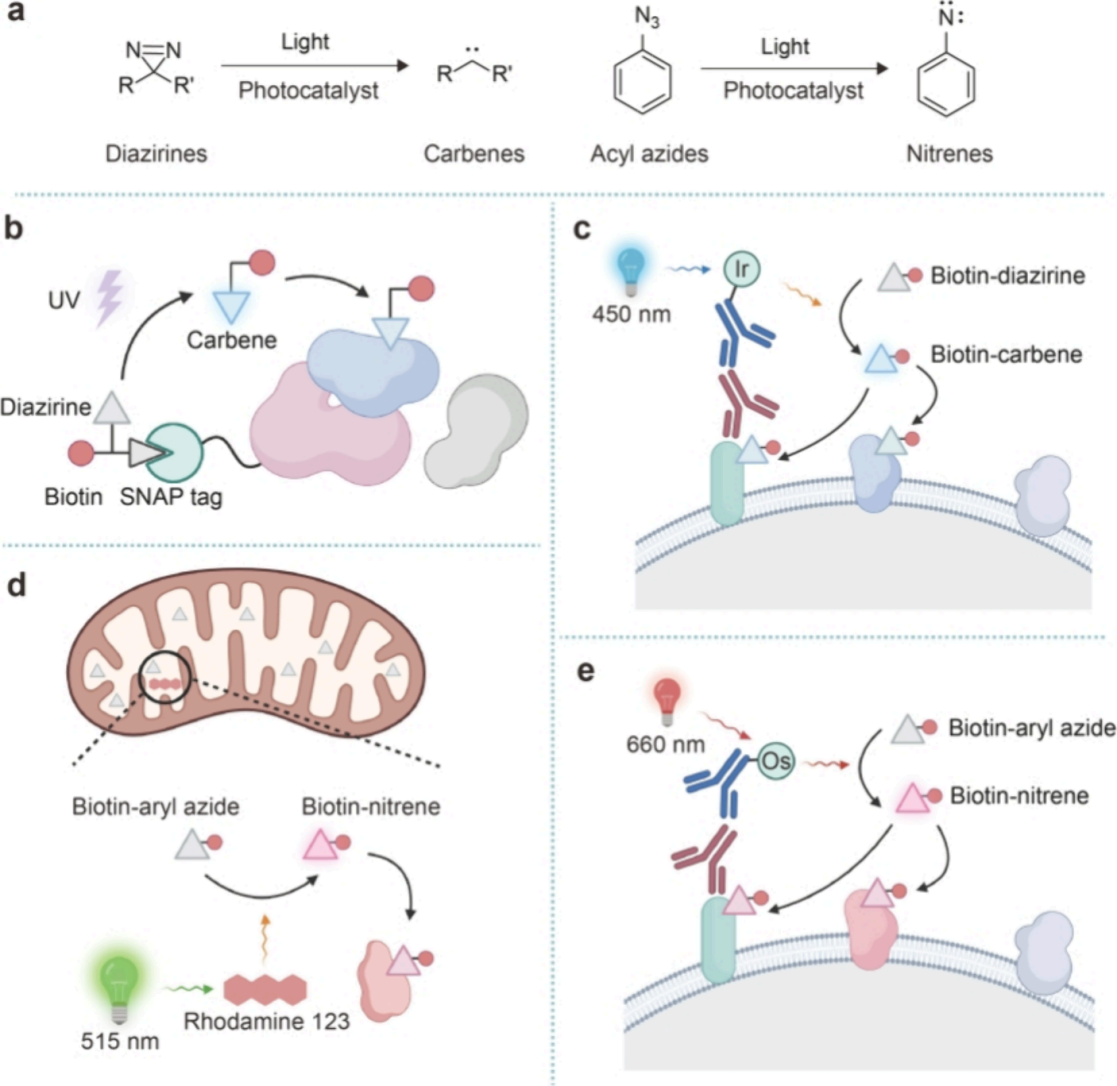
Proximitomics based on carbenes and nitrenes. (a) Upon activation by a photocatalyst, diazirines and aryl azides are converted into carbenes and nitrenes, respectively. (b) PhotoPPI utilizes a trifunctional probe that introduces freely diffusible carbenes to capture protein interaction networks. (c) μMAP uses an iridium-based photocatalyst to activate diazirines into carbenes upon 450 nm light irradiation. The generated carbenes thereby covalently tag proximal proteins for analysis. (d) Rhodamine 123 acts as a photocatalyst that converts aryl azides into nitrenes upon 515 nm light irradiation for mitochondrial proteomic analysis. (e) An osmium-based photocatalyst enables the generation of nitrenes from aryl azides under deep-red light irradiation.

Carbenes and nitrenes are neutral and highly reactive species, which contain divalent carbon and monovalent nitrogen atoms, respectively. They are both surrounded by a sextet of electrons and can exist in a singlet or a triplet state. Carbenes and nitrenes can react with nearby biomolecules with extremely high kinetics and form covalent linkages via multiple mechanisms such as direct insertion of X–H bonds (X = C, O, N, etc.).^67^ Upon UV light irradiation, carbenes and nitrenes are generated from diazirines and aryl azides, respectively, making them commonly used reagents for labeling and cross-linking of biomacromolecules (Figure 5a).^68−70^ For example, cross-linkers using NHS ester and diazirine as the warheads have been employed for capture of protein–protein and protein–RNA interactions.^71,72^ Moreover, the genetic code expansion technique enabled site-specific incorporation of unnatural amino acids bearing a diazirine moiety into a protein of interest (POI), thus allowing for mapping of the protein interaction network in living cells.^73,74^ In addition, drugs and small-molecule ligands modified with a diazirine or an aryl azide serve as photoaffinity labeling reagents for identifying the binding proteins in living cells.^75^ These applications highlight that carbenes and nitrenes are promising reactive species for proximity labeling.

To exploit carbenes and nitrenes for proximitomics, they need to be not only generated locally but also able to diffuse freely. One strategy is anchoring the precursors onto specific proteins for localization, followed by the photoactivation of carbenes/nitrenes with simultaneous release from the proteins. In 2019, the Moellering group demonstrated such a method, termed photoproximity protein interaction (PhotoPPI) profiling (Figure 5b).^76^ To construct the chemical probe of PhotoPPI, a benzylguanine (BnG) moiety that can be recognized by and covalently attached to SNAP-Tag fused on the POI is linked to a diazirine–biotin conjugate via a photocleavable linker. Upon illumination with 365 nm UV light, the diazirine is activated into carbene, and at the same time the probe is released from the SNAP-POI and covalently label proximal proteins. Of note, the carbene was generated as a stoichiometric product, limiting the labeling efficiency.

To overcome this limitation, the groups of MacMillan, Oslund, and Fadeyi collaboratively developed the MicroMap (μMap) strategy, which pioneered the use of photocatalyst to generate carbenes in living systems (Figure 5c).^77^ By using an iridium photocatalyst–antibody conjugate to spatially localize carbene generation, μMap enabled the selective labeling of proteins proximal to the POI with high spatial resolution. Chemically, the iridium photocatalyst is activated by blue light, triggering a Dexter energy transfer and activating nearby diazirines into a T_1_ state, followed by elimination of nitrogen and carbene generation. The blue light cannot directly activate diazirines, ensuring a high spatial resolution. Furthermore, as water can rapidly quench carbenes, μMap presents the highest spatial resolution compared with other proximity labeling strategies, making it well-suited for mapping protein–protein interactions on the cell surface.

Similar to μMap, the photocatalytic generation of nitrenes has also enabled proximitomics. In 2021, the Chen and Zhang groups demonstrated that organic small-molecule dyes could serve as photocatalysts for generating triplet nitrenes from aryl azides under visible light irradiation (Figure 5d).^78^ By employing rhodamine 123 that targeted mitochondria in combination with aryl azide–biotin, specific labeling and proteomics analysis of mitochondrial proteins were performed. Recently, the groups of Rovis, Fadeyi, and Oslund developed an osmium-based photocatalyst that activated aryl azide probes into triplet nitrenes under deep-red light (λ = 660 nm) for protein proximity labeling on cell surfaces (Figure 5e).^79^ The use of light with a longer wavelength reduces background activation of photoactive small-molecule probes and thus improves the efficacy in complex biological environments. Of note, these small-molecule photocatalysts have not been targeted to specific subcellular regions via genetic engineering. Such methods, once developed, would greatly expand the applications of carbenes and nitrenes in intracellular proximity labeling.

### 2.5 Quinone Methides

**Figure 6:**
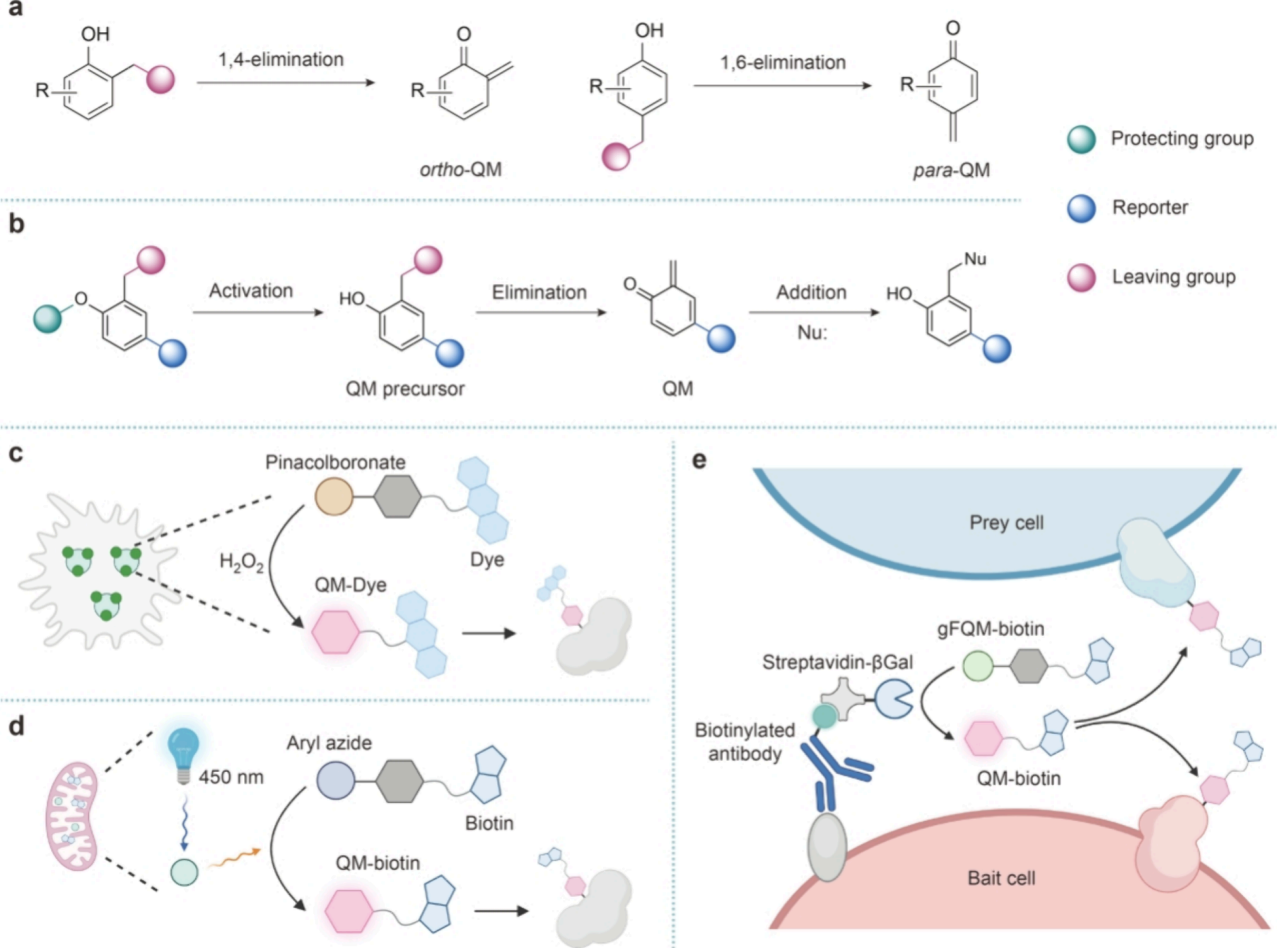
Proximitomics based on QM. (a) QMs are generated via 1,4- or 1,6-elimination from the corresponding phenol precursors bearing a halomethyl group at the ortho- or para-position, respectively. (b) Upon activation, the protecting group is cleaved to generate QM precursors and QMs, which rapidly tag nearby nucleophiles via Michael addition. (c) A pinacolboronate group is cleaved upon H_2_O_2_ oxidation, followed by the generation of QM–biotin for subcellular proteome mapping. (d) A mitochondria-localized photocatalyst is utilized to decage the aryl azide group in a QM probe. Followed by elimination and QM formation, mitochondrial proteins are selectively labeled and analyzed. (e) A membrane-tethered β-galactosidase is used to activate the QM probe protected by a β-galactosyl group. The generated QMs covalently tag nearby cells for analyzing the cell spatial organization in tissue samples.

Quinone methides (QMs) are a class of highly electrophilic species that can be generated via 1,4- or 1,6-elimination from the corresponding phenol precursors bearing a halomethyl group at the ortho- or para-position, respectively (Figure 6a).^80^ Once generated, QMs rapidly react with various nucleophiles in biomolecules via Michael addition, resulting in the formation of QM adducts. Since the phenolic hydroxyl group can be chemically protected or caged, the formation of QMs can be triggered by a variety of means, such as enzymatic decaging and photoactivation (Figure 6b).^81,82^ This allows for precise control of QM formation and the subsequent labeling of proximal biomolecules, which has led to a series of studies using QM as enzyme inhibitors,^83^ fluorophore immobilizers,^84^ and reactivity warheads for enzyme imaging and activity-based labeling.^85,86^

It has recently been recognized that QMs meet all of the criteria of reactive species for proximitomics. In comparison to other reactive species, activation of QMs can be readily achieved via a list of mechanisms that enables proximitomics in systems not amenable to other reactive species. For example, the Hamachi group developed the H_2_O_2_-responsive QM probe Hyp-L to selectively identify the proteome in the H_2_O_2_-enriched subcellular regions (Figure 6c).^87^ The oxidative conversion of the pinacolboronate group to the phenolic hydroxyl group by H_2_O_2_ generated a QM precursor from which the subsequent elimination step produced QMs for labeling nearby proteins. Recently, the same group further demonstrated Cu(I)-activated QM formation and proximity protein labeling.^88^ These works highlighted the applications of QMs in proximity labeling triggered by small molecules. In 2021, the Chen group introduced the CAT-Prox method for identifying mitochondrial proteins (Figure 6d).^89^ CAT-Prox utilized a mitochondria-localized photocatalyst that catalyzed the decaging of the aryl azide group in the PAB-QM–biotin probe upon blue light illumination, generating QMs in situ. Mitochondrial proteins were thus labeled with QM probes. Subsequently, the same group expanded this strategy to cell surfaces by employing antibody–photocatalyst conjugates for surfaceome identification.^90^

Although the aforementioned studies suggest that the labeling radius of QMs is sufficiently short to ensure subcellular proteome mapping (e.g., less than 1 μm for Hyp-L), earlier investigations revealed the half-lives of QMs in aqueous solutions are actually at the second level, indicating that the QMs are more suitable for proximitomics at larger scales.^91^ Capitalizing on this property of QMs, our group developed quinone methide-assisted identification of cell spatial organization (QMID), a novel chemical method for cellular-scale proximity labeling (Figure 6e).^92^ QMID was built on the combination of a caged QM probe and a decaging enzyme. By displaying the decaging enzyme on the surface of specific cell types, the QM probe was decaged in situ and rapidly labeled proximal cells for downstream analysis. An antibody-conjugated β-galactosidase (βGal) was used to activate the QM probe, protected by a β-galactosyl group. As the first example of cellular-scale proximitomics in tissue samples, QMID was applied to the discovery of distinctive cell populations and gene expression patterns in the immune niches of specific T cell subtypes in the mouse spleen.

In contrast to other reactive species, a notable feature of QMs is their adaptability to diverse chemical modifications. This property offers the potential for fine-tuning the reactivity, half-life, and consequently the labeling radius of QMs. It is worth noting that the generation of QMs involves three sequential steps: (i) deprotection of the caged phenol to generate a QM precursor, (ii) self-elimination of the QM precursor to form a QM, and (iii) Michael addition of QM with biomolecules. Given that the diffusion of the QM precursor and QM itself collectively determines the labeling radius, which may be oppositely influenced by the chemical modifications in the QM structure, careful evaluations are needed for designing new QM probes. For instance, previous studies have demonstrated that electron-withdrawing groups serve to stabilize the QM precursor while accelerating the Michael addition of QM.^93^ Conversely, electron-donating groups facilitate the self-elimination of the QM precursor but decelerate the formation of QM adducts. Recently, the Chen group evaluated the reactivity of a panel of QM structures and found a better warhead with improved protein labeling intensity.^94^ Further screening is needed to identify new QM structures for proximity labeling on different scales.

## 3 Conclusion and Perspectives

Harnessing proximity labeling based on in situ activated reactive species, proximitomics delves into the spatial organization of biomolecules and cells, thereby facilitating a comprehensive analysis of their functional interactions. A collective comparison of the currently available reactive species is provided in this work (Table 2). Since most of the reactive species exhibit labeling radii of nanometers, initial proximitomics studies were mainly performed at the molecular scale. To date, proximitomics has emerged as a widely used approach for deciphering the molecule–molecule interaction (MMI) networks, including protein–protein, protein–RNA, and protein–DNA interactions.^5^ In these applications, the activator of reactive species is directed to a bait protein/RNA/genomic locus, initiating proximity labeling to capture neighboring biomolecules for subsequent molecular identifications. Of note, the diffusion of activated reactive species leads to the labeling of not only direct binders but also biomolecules in proximity but not directly interacting. As a result, setting proper controls (e.g., a spatial reference control) becomes crucial for mitigating false positives. In addition to profiling molecular interactions, molecular-scale proximitomics also facilitates subcellular omics analysis, including subcellular proteomics and transcriptomics, by directing the activators into specific subcellular compartments to capture all the biomolecules within the region.^95,96^ Moreover, the recombinant expression of activators controlled by cell-type-specific promoters allows for cell-selective subcellular omics. The use of spilt versions of activators further enables proximity labeling occurring only at the interfaces of organelles or cells, thus eliminating background labeling. Of note, reactive species with larger labeling radii are expected to introduce more false positives, making proximity labeling with singlet oxygen, carbenes, and nitrenes particularly suitable for membraneless structures and cell surfaces. Of the discussed reactive species, all are reactive to proteins with distinctive preferences for amino acid residues. Notably, activated carboxylates stand out as the only species that are chemically inert to nucleic acids. Nevertheless, the use of carbenes, nitrenes, and quinone methides has been confined to protein labeling so far. Further exploration of these reactive species for proximity labeling of nucleic acids is of great interest.

**Table 2:** Comparison of the Reactive Species for Proximitomics

pecies	amenable to modification	activator	substrate	labeling radius^b^	in vivo feasibility	application scenario^c^
phenoxyl radicals	yes	enzymes and photocatalysts	proteins (Tyr) and nucleic acids (G)	∼20 nm (270 nm)	no	MMI, subcellular omics, CCI
activated carboxylates	no	enzymes	enzymes	∼10 nm	yes	MMI, subcellular omics
singlet oxygen	yes^a^	photocatalysts	proteins (Cys, His, Tyr) and nucleic acids (G)	∼30 nm	no	MMI, subcellular omics, CCI
carbenes	yes	only small-molecule photocatalysts	all residues in proteins and nucleic acids	<4 nm (50–120 nm)	no	MMI, subcellular omics, CCI
quinone methides	yes	proteins (Arg, Cys, Lys, Ser, Glu, and Asp) and nucleic acids (A, C, and G)	MMI, subcellular omics	<1 μm (micrometers)	yes	subcellular omics, cell spatial organization

a Chemical modification is made on the amine probes.

b Extracellular labeling radius is presented in parentheses.

c MMI, molecule–molecule interactions including protein–protein, protein–RNA, and protein–DNA interactions. CCI, cell–cell interactions.

Beyond MMIs, proximitomics also greatly facilitates the functional analysis of interactions and the spatial organization of cells. In cellular-scale proximitomics, the activator is typically deployed on the surface of bait cells so that proximity labeling occurs at the cell–cell interface and marks both the bait and interacting prey cells for subsequent analysis. Representative examples include μMAP,^77^ PhoTag,^28^ and PhoXCELL,^60^ which utilize carbenes, phenoxyl radicals, and singlet oxygen as the reactive species. Their extremely high reactivity leads to a diffusion and labeling radius of approximately 50–250 nm. As a result, only interacting cells with direct contact are recorded in these strategies, making them valuable tools for analyzing dynamic and transient cell–cell interaction systems such as the interactions between antigen-presenting cells (APC) and T cells. However, these approaches fall short of ideal choices for studying cell spatial organization since the short labeling radii limit their efficacy in capturing interactions among cells without direct contact. By using QMs as the reactive species that have a labeling radius of micrometers, the QMID technique solves this issue and allows for mapping cell spatial organization in mouse tissue samples.^92^ A promising future direction is to develop cellular-scale proximity labeling methods to record the cell spatial organization of multiple cell layers, which will better elucidate the cellular niche of rare cell types.

As discussed, the three hierarchical levels of proximitomics—MMI, subcellular omics, and CCI—hinge on the labeling radius of reactive species, which is dependent on the chemical reactivity and the concentrations of substrates and quenchers in different biological contexts. For example, the labeling radius of phenoxyl radicals is markedly influenced by the concentration of quenchers in the surrounding environment. Inside the cells, phenoxyl radicals are readily quenched by abundant cellular reductants such as glutathione, resulting in a labeling radius less than 20 nm.^11^ By contrast, the concentration of potential quenchers is substantially lower in the extracellular space, resulting in a larger labeling radius of about 250 nm.^97^ Considering that the bioAMP anhydride preferentially reacts with primary and secondary amines, proteins and free amino acids should be the major sources of substrates and quenchers. Although it has not been precisely measured, a larger labeling radius of bioAMP in extracellular environments is expected. Similarly, the half-life and labeling radius of ^1^O_2_ have not been quantitatively determined either. Nevertheless, the labeling radius of ^1^O_2_ in the extracellular space appears to be short enough to allow for contact-dependent labeling of cell–cell interactions, as demonstrated by PhoXCELL.^60^ A recent work, however, indicated a cellular-level labeling radius for ^1^O_2_ in the detection of cell–cell interactions.^98^ This discrepancy highlights the need for further investigations into the kinetics and labeling radius of ^1^O_2_-based proximity labeling, particularly for extracellular applications. As carbenes are highly reactive, they are rapidly quenched by water, rendering them very short labeling radii. Consequently, the labeling radius of carbenes is believed to be similar regardless of the biological contexts. For QMs with the lowest reactivity among these reactive species, they are probably not well-suited for MMI studies but cover the applications from subcellular omics to CCI. Similar to phenoxyl and bioAMP, QMs should have larger labeling radii extracellularly than the ones inside the cells since nucleophiles that quench QMs are more concentrated intracellularly.

For a long time, proximitomic analysis has been confined to cultured cells. It is now well-recognized that in vivo proximitomics that analyzes molecular and cellular interactions in their native contexts is of great importance. Notably, not all of the reactive species and the corresponding proximity labeling methods are compatible with in vivo applications. For example, activation of phenoxyl radicals, singlet oxygen, and carbenes relies on either cytotoxic H_2_O_2_ or light illumination with short wavelength and poor tissue penetration, making them not applicable in vivo. Although recent studies demonstrated the activation of nitrenes using a combination of metal photocatalysts and deep-red light that has a better tissue penetration,^79^ in vivo delivery of photocatalysts also remains a challenge. Alternatively, reactive species that can be activated by an enzyme and endogenous reagents offer more promising options for in vivo proximitomics. This includes activated carboxylates and quinone methides, which can be generated by variants of biotin ligase and a set of decaging enzymes, respectively. A compelling future direction is to develop new enzymes capable of activating reactive species (e.g., carbenes and phenoxyl radicals) without the use of light or cytotoxic reagents, which will greatly facilitate the use of other reactive species in vivo. Finally, beyond the existing toolbox, other chemical species should be continuously explored as reactive species for proximitomics. For example, two recent studies demonstrated the use of tyrosinase and esterase to generate benzoquinone and acyl chloride, respectively, for molecular-scale proximitomics.^99,100^
